# Optimization of culture medium to improve bio-cementation effect based on response surface method

**DOI:** 10.1038/s41598-024-58063-1

**Published:** 2024-04-16

**Authors:** Zhikun Pan, Shiding Cao

**Affiliations:** 1 Shenzhen SEZ Construction Solid Waste Resources Co. Ltd., Shenzhen, 518034 China; 2 State Key Laboratory for Tunnel Engineering, China University of Mining and Technology Beijing, Beijing, 100083 China; 3 Shenzhen General Integrated Transportation and Municipal Engineering Design and Research Institute Co. Ltd., Shenzhen, 518003 China

**Keywords:** Microbially induced calcite precipitation, Prediction model, Response surface method, Bio-cementation, Biofilm growth, Biochemistry, Biogeochemistry

## Abstract

The main challenge in the large-scale application of MICP lies in its low efficiency and promoting biofilm growth can effectively address this problem. In the present study, a prediction model was proposed using the response surface method. With the prediction model, optimum concentrations of nutrients in the medium can be obtained. Moreover, the optimized medium was compared with other media via bio-cementation tests. The results show that this prediction model was accurate and effective, and the predicted results were close to the measured results. By using the prediction model, the optimized culture media was determined (20.0 g/l yeast extract, 10.0 g/l polypeptone, 5.0 g/l ammonium sulfate, and 10.0 g/l NaCl). Furthermore, the optimized medium significantly promoted the growth of biofilm compared to other media. In the medium, the effect of polypeptone on biofilm growth was smaller than the effect of yeast extract and increasing the concentration of polypeptone was not beneficial in promoting biofilm growth. In addition, the sand column solidified with the optimized medium had the highest strength and the largest calcium carbonate contents. The prediction model represents a platform technology that leverages culture medium to impart novel sensing, adjustive, and responsive multifunctionality to structural materials in the civil engineering and material engineering fields.

## Introduction

In recent years, bio-cementation or microbially induced carbonate precipitation (MICP) has garnered significant attention from researchers in the fields of geotechnical and environmental engineering^1–4^. The MICP technique operates on the principle that metal ions bind with acid radical ions to form minerals, such as calcium carbonate (CaCO_3_), which possess cementing properties^5,6^. Among the various pathways available, the hydrolysis of urea by urease-producing bacteria stands out as one of the most widely adopted methods^7,8^. The potential for implementing MICP is widely acknowledged^9–13^, encompassing benefits such as enhanced soil strength and stiffness^2^, reduced soil permeability^14^, crack repair capabilities^15,16^, improved mechanical properties of granular soils^16^, and erosion control measures^17–19^.

The precipitation of CaCO_3_ can facilitate the formation of bridges between granular soil particles, resulting in the cementation and improved cohesion of loose soil particles. Despite extensive research on soil improvement using MICP, limitations arise when applying this method to bond coarser materials. The cost-effectiveness of MICP treatment decreases for coarse materials due to the requirement for a higher number of biochemical treatment cycles and a larger amount of cementitious material to achieve acceptable strength^20^. However, given that coarse materials are commonly encountered in practical engineering applications, the widespread implementation of MICP is hindered.

The main challenge in the large-scale application of MICP lies in its low efficiency and promoting biofilm growth can effectively address this problem. If the bonding points between coarse particles are effectively cemented, the sample would demonstrate significant solidification effects. To enhance the cementation effect at these bonding points, rapid formation and growth of biofilm is essential^21,22^. Precipitated CaCO_3_ becomes attached to the biofilm, resulting in an increased amount of CaCO_3_ precipitation available at the bonding points as the biofilm rapidly forms and grows, ultimately leading to improved cementation effects^23–26^. However, limited research has been conducted on the rapid growth of biofilm in bio-cementation treatment. Previous studies have utilized yeast extract^20^, peptone^4^, and ammonium sulfate^27^ as commonly employed nutritional sources for cultivating urease-producing bacteria. Additionally, other available nutritional sources include nutrient broth^3,28^ and glucose^15^. The growth of biofilms is closely associated with the nutrient composition of the culture medium; however, limited research has been conducted on investigating the impact of different nutritional sources on biofilm growth and bacterial cell performance.

Therefore, in the present study, a prediction model utilizing the Response Surface Method (RSM) was proposed to optimize the culture medium and achieve the desired biomass concentration and growth rate by considering the impact of nutrients on biofilm growth and bacterial cell performance. Subsequently, bio-cementation tests were conducted on sands using the optimized medium and several other culture media. The unconfined compressive strength (UCS) test, biofilm growth, and quantification of CaCO_3_ were employed as indicators to evaluate the effects of bio-cementation. A comparison of the cementation effects demonstrated the advantages of the optimized culture medium and confirmed the feasibility of the prediction model. This predictive model would enable precise control over the culture medium for materials with diverse pore sizes, facilitating on-demand sensing, adjustment, growth, biomineralization, and subsequent solidification of building materials. Consequently, this prediction model opens up novel possibilities for material manufacturing and utilization within civil engineering and material engineering domains.

## Materials and methods

### Bacteria and Enzyme Activity

In this study, we employed a strain of ureolytic bacteria known as *Sporosarcina pasteurii* (*S. pasteurii*; ATCC 11859). The changes in cell density were assessed by monitoring the absorbance (optical density) of the suspension at a wavelength of 600 nm (OD_600_)^6,29^. Therefore, optical density (OD_600_) was utilized to represent the cell density in this paper.

According to the methodology proposed by^30^, a bacterial suspension of 6 ml was mixed with a urea solution (1 mol/l) of 54 ml, and subsequently, electrical conductivity measurements were taken every 5 min. The average change in conductivity per minute (ms/cm∙min) was calculated, corresponding to an experimental determination of 11 mM urea hydrolyzed/min^30^. Therefore, the change in conductivity per minute (ms/min) can be converted into the amount of urease hydrolysis over a specific time period. Ultimately, by multiplying this value by the dilution factor of 10, the rate of urea hydrolysis per minute (mM/min), representing enzyme activity, was obtained.

### Response surface method

The following discusses three key aspects of the RSM: the spatial distribution of sample points, the degree of polynomial for response surface modeling, and the estimation of failure probability.

Firstly, a response surface provides a localized approximation of the limit state function within the vicinity of the sample points, thereby making the accuracy of this approximation dependent on the distance between these points. Naturally, the precision is also influenced by both the nature of the limit state function and the response surface employed. For simplicity, let us consider a quadratic response surface without mixed terms^31^. The sample points in the U-space for standard normal variables are positioned at the center of an experimental design as well as along its coordinate axes. The parameter f denotes the distance between these central and other points. If f assumes a large value, then it implies that distant points in the U-space are being interpolated by the response surface. Consequently, fluctuations in the limit state function between these points may not be accurately reproduced. Conversely, when the value of f is low, the response surface can accurately represent only a limited portion of the limit state function (LSF). Moreover, as f decreases, the system of equations tends to become ill-conditioned^32^. Therefore, selecting an appropriate value for f depends on the non-linearity of the involved limit state function in the reliability problem. Consequently, solving a reliability problem should also incorporate sensitivity analysis regarding parameter f.

The second observation pertains to the polynomial employed in approximating the limit state function (LSF). The selection of the degree of the response surface should strike a balance between reliability estimate accuracy and analysis efficiency. In terms of accuracy, opting for a higher degree polynomial allows for a remarkable approximation of the LSF. To achieve an accurate estimation of failure probability, it is crucial to focus on accurately approximating the LSF near the design point as this region contributes significantly to failure probability. Therefore, coupling the use of response surfaces with an optimization algorithm becomes essential in locating the design point. Subsequently, estimating failure probability can be accomplished through importance sampling based on said design point^33^.

### Culture media optimization

In order to enhance biomass growth, the culture medium was optimized by investigating the impact of nutrients on the growth of *S. Pasteurii*. Previous studies have commonly employed yeast extract, polypeptone, and ammonium sulfate as nutritional sources for cultivating *S. Pasteurii*^4,20,27^. Therefore, a combination of RSM and Central Composite Design (CCD) was applied to determine the optimal concentrations of yeast extract, polypeptone, and ammonium sulfate in the culture medium. Additionally, 10.0 g/l of NaCl was included in the medium to ensure sufficient salt content for bacterial growth while maintaining an initial pH value of 7.0.

**Figure 1:**
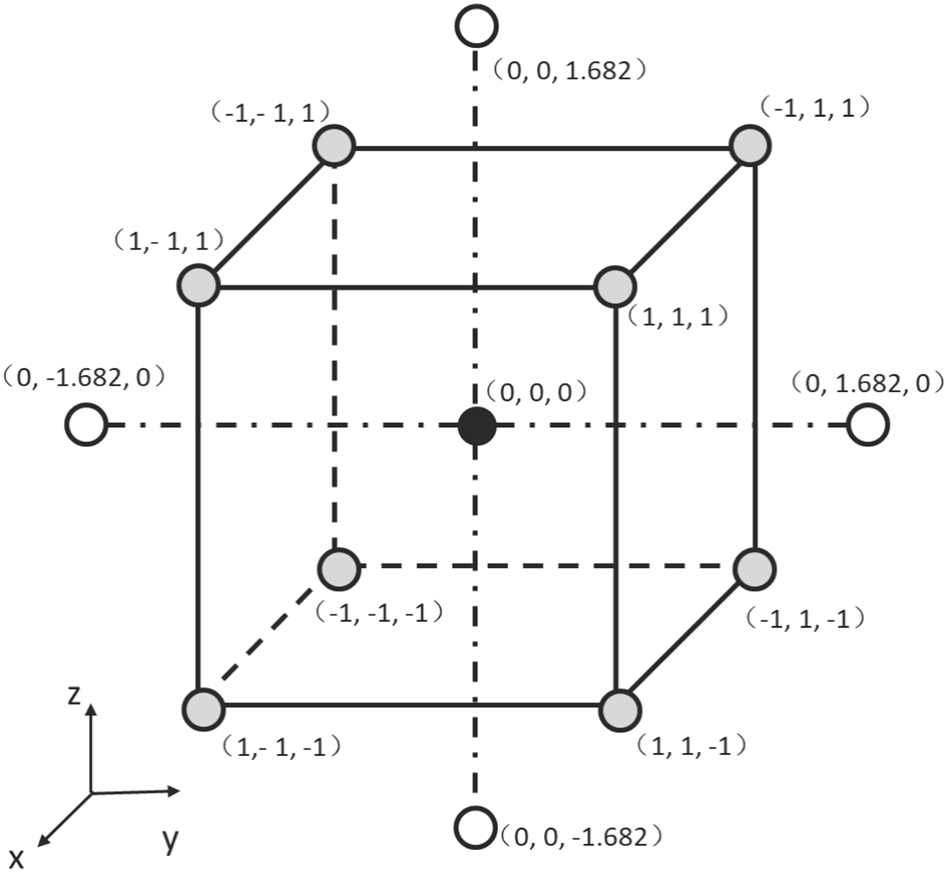
Variables coding in CCD with three parameters.

The consideration of three factors in CCD required the utilization of five coded levels (−a, −1, 0, + 1, and + a), as illustrated in Fig. 1. A polynomial model (Eq. 1) was employed to accurately represent the optical density (OD_600_).

1 $$Y={a}_{0}+\sum_{i=\mathrm{1,2},3}{a}_{i}{x}_{i}+\sum_{i=\mathrm{1,2},3}{a}_{ii}{{x}_{i}}^{2}+\sum_{i,j=\mathrm{1,2},3}{a}_{ij}{x}_{i}{x}_{j}+c$$

where $Y$ is the predicted result determined by the coded variables of yeast extract, polypeptone, and ammonium sulfate; ${x}_{i}$, is the coded variable of yeast extract, polypeptone, or ammonium sulfate; ${a}_{0}$ is the intercept term; ${a}_{i}$ is the linear coefficients; ${a}_{ii}$ is the quadratic coefficients; ${a}_{ij}$ is the interaction coefficients, and c is the error value of prediction model.

The data obtained in this study were optimized and analyzed using Minitab (version 17.1; Minitab Inc., Pennsylvania, USA). Table 1 presents the coded levels of the three factors based on the CCD method and the selected parameter values for five coded levels. Subsequently, a predictive model for culture medium was developed based on OD_600_ measurements.

**Table 1:** Coded values of three factors and measured optical density at a wavelength of 600 nm (OD_600_).

Run no.	Yeast extract (× 1)	Polypeptone (× 2)	Ammonium sulfate (× 3)	Measured optical density (OD_600_)
1	−1.000	1.000	−1.000	1.38
2	1.682	0.000	0.000	1.87
3	0.000	0.000	0.000	0.91
4	−1.000	1.000	1.000	0.45
5	0.000	0.000	0.000	0.91
6	0.000	0.000	0.000	0.91
7	0.000	0.000	1.682	0.29
8	−1.000	−1.000	1.000	0.37
9	0.000	0.000	−1.682	0.99
10	0.000	0.000	0.000	0.91
11	−1.000	−1.000	−1.000	1.21
12	1.000	−1.000	−1.000	1.39
13	−1.682	0.000	0.000	0.25
14	0.000	1.682	0.000	1.17
15	1.000	−1.000	1.000	0.81
16	1.000	1.000	−1.000	1.98
17	0.000	0.000	0.000	0.91
18	0.000	−1.682	0.000	0.41
19	1.000	1.000	1.000	0.72
20	0.000	0.000	0.000	0.91

Coded level −1.682 corresponds to 2.97 g/l; coded level −1.000 corresponds to 5.00 g/l; coded level 0.000 corresponds to 12.50 g/l; coded level 1.000 corresponds to 20.00 g/l; coded level 1.682 corresponds to 33.63 g/l.

### Sand solidification

#### Sands and MICP treatment

**Figure 2:**
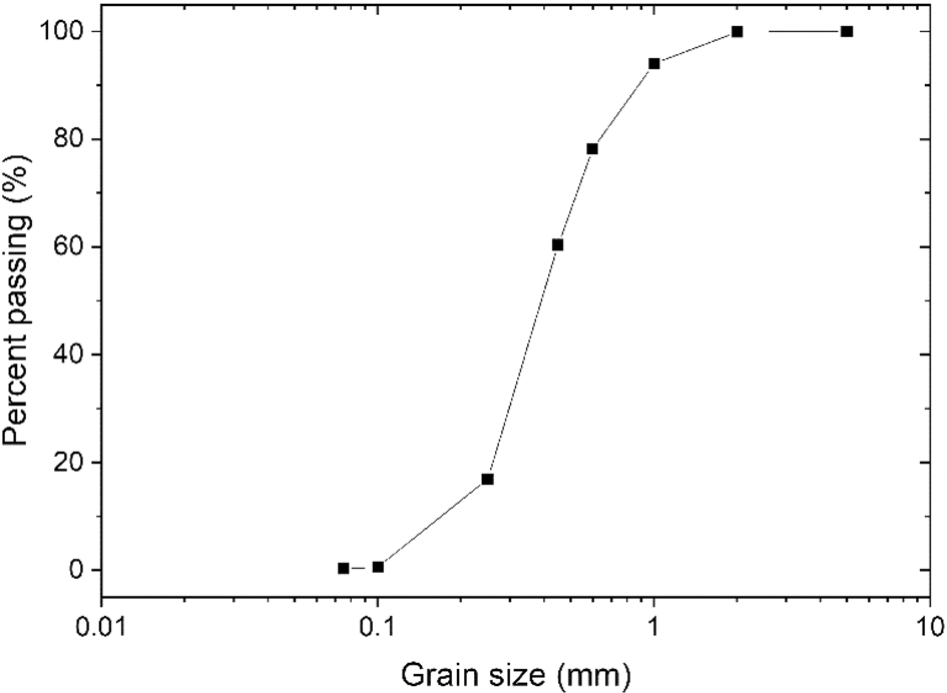
Grading curve of sands.

The gradation of the sand utilized in the sand solidification experiment is shown in Fig. 2. The sand exhibited inadequate gradation, and according to the USCS classification system^34^, it was categorized as SP due to its median diameter (D_50_) measuring 0.32 mm. All sand columns possessed an identical initial dry density of 1.61 g/cm^3^, with an inner diameter of 5.0 cm and a height of 10 cm.

Firstly, a bacterial suspension of 40 mL was injected into the specimens from the bottom at a controlled rate of 8 mL/min using an electric pump. The samples were allowed to incubate for 2 h, after which a gelling solution (consisting of 1.0 M urea and calcium chloride) was injected in a volume of 40 mL. The initial pH value of the gelling solution was maintained at 7.0 throughout the experiment. To ensure efficient experimentation, this treatment cycle was repeated every 48 h for a total of six biochemical treatment cycles.

#### Evaluation of treatment effects

After a 12-day period of biochemical treatment, UCS tests were conducted in accordance with ASTM C617^35^ to evaluate the cementation effect of the samples. The loading speed was maintained at a constant rate of 1 mm/min throughout the UCS tests.

The optimized medium was determined using a prediction model to achieve rapid biofilm growth. Subsequently, the concentration of nutrients (yeast extract, polypeptone, and ammonium sulfate) in the culture medium was varied to obtain different media for comparison. The correlation between viable cell count and optical density (OD_600_) was examined, establishing the reliability of OD_600_ measurements and enabling the estimation of confirmatory viable bacterial cell numbers under each condition. To evaluate the biofilm growth rate across different media, the treated sand particles with a similar amount were sampled and then immersed in the LB medium. After that, sand particles were stirred to easily scrape the biofilm between sand particles. The plate colony counting method was used again to determine colony forming units (CFU). This method was used to evaluate the biofilm growth rate related to the viable bacteria, which can be easily obtained via the above-mentioned scraping method. In addition, the same method was used to obtain the biofilm in sand particles treated with different media; thus, the biofilm data were comparable.

Additionally, the CaCO_3_ content was determined through gravimetric acid washing (2 M HCl) technique^30^. Subsamples were dried at 70 °C for 48 h and weighed before and after acid wash. The difference in weight indicated the mass of precipitated CaCO_3_, which was then divided by the soil mass to obtain the CaCO_3_ content.

## Results and discussion

### Significance and evaluation of prediction model

The optical density values at 48 h in Table 1 were utilized as the measured outcomes in the CCD test. Through Minitab analysis of the measured optical density (OD_600_), coefficients within the polynomial model were derived, as presented in Eq. (2).

2 $$Y=0.903+0.3086{x}_{1}+0.1485{x}_{2}-0.3505{x}_{3}+0.0981{{x}_{1}}^{2}+0.0026{{x}_{2}}^{2}-0.0504{{x}_{3}}^{2}+0.0312{x}_{1}{x}_{2}-0.0087{x}_{1}{x}_{3}-0.0963{x}_{2}{x}_{3}$$

**Figure 3:**
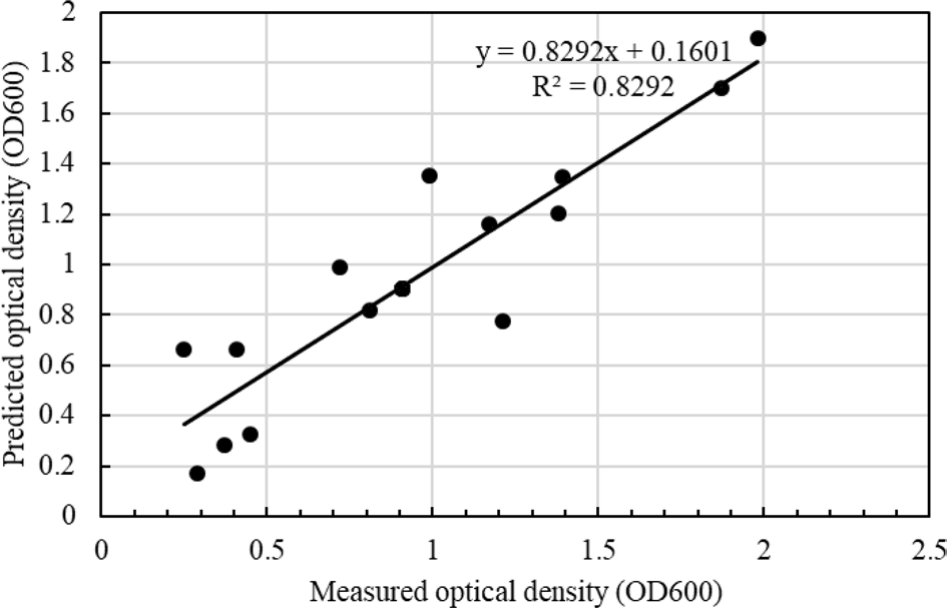
Predicted values of optical density against the measured results.

Based on Eq. (2), the relationship between the coded values of the parameters and optical density (OD_600_) was investigated. An analysis of variance (ANOVA) was conducted (Table 2) to assess the agreement between measured and predicted results^36^. The comparison between predicted and measured outcomes is presented in Fig. 3, demonstrating a close alignment. In the CCD test, an R^2^ coefficient of 82.92% indicated that the proposed prediction model accounted for 82.92% variation in the dependent variable. While considering only nutrients in the medium within our prediction model, we overlooked any potential impact of salt content on bacterial growth, resulting in an R^2^ value lower than that reported in^37^ at 90.21%. In general, a model with a high fitting degree should have a larger R^2^ value in the RSM, which should be over 60%^38,39^. Therefore, the R^2^ value of 82.92% that was obtained in the present study still proved the validity of the proposed prediction model.

**Table 2:** Evaluation of the prediction model.

Source	DF	Adj SS	Adj MS	F-value	P-value
Model	9	3.55382	0.39487	5.39	0.007
Linear	3	3.27991	1.09330	14.93	0.001
Yeast extract	1	1.30060	1.30060	17.76	0.002
Polypeptone	1	0.30120	0.30120	4.11	0.070
Ammonium sulfate	1	1.67812	1.67812	22.92	0.001
Square	3	0.19137	0.06379	0.87	0.488
Yeast extract × yeast extract	1	0.13871	0.13871	1.89	0.199
Polypeptone × polypeptone	1	0.00010	0.00010	0.00	0.971
Ammonium sulfate × ammonium sulfate	1	0.03658	0.03658	0.50	0.496
2-way interaction	3	0.08254	0.02751	0.38	0.772
Yeast extract × polypeptone	1	0.00781	0.00781	0.11	0.751
Yeast extract × ammonium sulfate	1	0.00061	0.00061	0.01	0.929
Polypeptone × ammonium sulfate	1	0.07411	0.07411	1.01	0.338
Error	10	0.73215	0.07322		
Lack-of-fit	5	0.73215	0.14643		
Pure error	5	0.00000	0.00000		
Total	19	4.28597			

S	R-sq	R-sq(adj)			
0.270583	82.92%	67.54%

In addition to the analysis of variance, the p-value serves as a crucial indicator in assessing the significance of parameters within the prediction model. A higher p-value suggests a parameter with less statistical significance^40^. Parameters that exhibit highly significant effects typically have p-values below 0.05. For parameters with p-values ranging from 0.05 to 0.1, they are considered marginally significant factors. Parameters with a p-value exceeding 0.1 do not significantly impact the prediction model^39^.

The p value of the prediction model was 0.007, as presented in Table 2, indicating its high significance and accurate predictive capability. Both yeast extract and ammonium sulfate exhibited highly significant parameters with p values of 0.001 each. Moreover, polypeptone demonstrated a marginally significant effect within the model (0.05 < p < 0.1). However, no statistically significant quadratic effects or interaction effects among yeast extract, polypeptone, and ammonium sulfate were observed.

### Mutual effects of parameters on optical density

**Figure 4:**
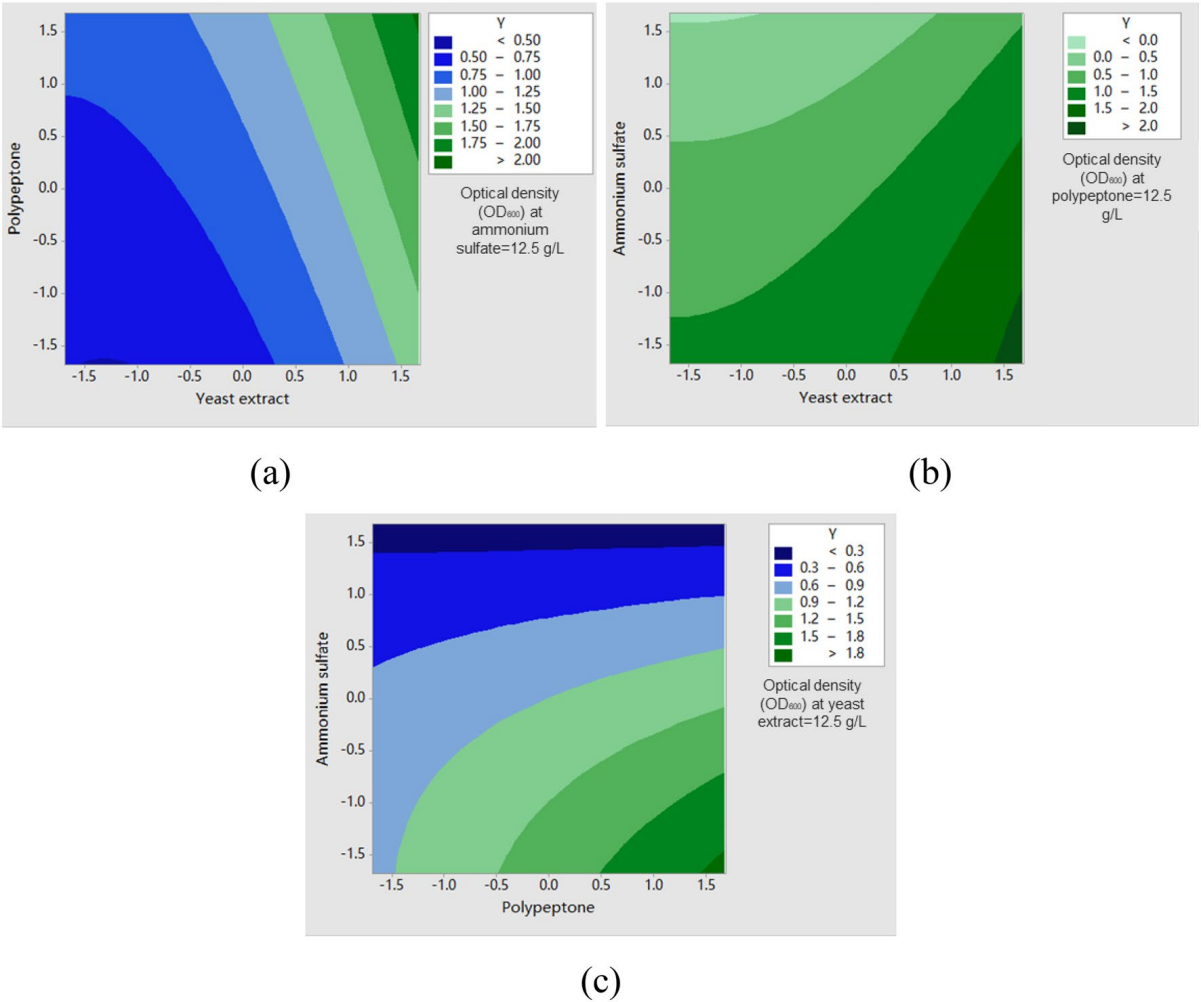
Response surface of optical density showing mutual interaction between the following: (**a**) yeast extract and polypeptone; (**b**) yeast extract and ammonium sulfate; (**c**) polypeptone and ammonium sulfate.

Furthermore, the interdependent effects of parameters on optical density (OD_600_) were investigated, and the findings are illustrated in Fig. 4. When ammonium sulfate was set at the central point (12.5 g/l), an increase in yeast extract concentration resulted in a more pronounced elevation in optical density (OD_600_) compared to that caused by polypeptone, as depicted in Fig. 4a. In other words, yeast extract exhibited greater significance than polypeptone. Conversely, when polypeptone was set at the central point (12.5 g/l), reducing ammonium sulfate concentration induced an increase in optical density (OD_600_). Notably, this increasing rate resembled that observed with yeast extract, as shown in Fig. 4b. A similar trend was also observed in Fig. 4c. With a yeast extract concentration of 12.5 g/l, decreasing ammonium sulfate concentration led to relatively higher growth rates in optical density (OD_600_). However, in the conducted experiment, the optical density (OD_600_) exhibited an initial increase followed by a subsequent decrease as the concentration of ammonium sulfate increased. In accordance with the prediction model, it is suggested that the optimal concentration of ammonium sulfate may be lower than the coded level of −1 (5 g/l), indicating that no initial increase occurred. Consequently, as the concentration of ammonium sulfate increased from 5 to 33.63 g/l, there was a gradual reduction in optical density (OD_600_), ultimately leading to a contradictory conclusion. The prediction model failed to account for the intricate effects of ammonium sulfate; nevertheless, its high significance ensures potential applications.

### Sand solidification

#### Culture media

The prediction model established the relationship between nutrient composition in the medium and bacterial growth, while examining the correlation between viable cell count and optical density (OD_600_). This analysis confirmed the reliability of optical density (OD_600_) measurements and allowed for the estimation of confirmatory viable bacterial cell numbers under each condition. Furthermore, the determination of the initial biomass growth rate was based on nutrient concentrations in the culture medium after bacterial cultivation. Consequently, the prediction model was employed to obtain an optimized medium with appropriate initial biomass concentration and growth rate post-cultivation.

According to the prediction model, the optimized medium was obtained by adjusting the initial nutrient concentrations in the medium (20 g/l yeast extract, 10 g/l polypeptone, and 5 g/l ammonium sulfate). In this model, with a combination of 20 g/l yeast extract, 10 g/l polypeptone, and 5 g/l ammonium sulfate, the predicted optical density (OD_600_) was determined to be 1.89, which exhibited reasonable agreement with the desired outcome. Notably, it was observed that for achieving optimal growth conditions, a lower concentration of ammonium sulfate than initially anticipated proved sufficient. Specifically, when no ammonium sulfate was added (0 g/l), the predicted optical density (OD_600_) remained close to the target value at 1.75. Consequently, in formulation A medium configuration described hereinabove did not include any addition of ammonium sulfate. According to the prediction model, the significance of polypeptone was found to be lower compared to yeast extract. When the concentration of polypeptone was increased from 10 to 20 g/l, a relatively smaller increment in optical density (OD_600_ = 2.08) was observed. Therefore, the concentration of polypeptone was adjusted to 20 g/l as medium B. By employing 33.63 g/l yeast extract, 33.63 g/l polypeptone, and no ammonium sulfate supplementation, the maximum optical density (OD_600_) achieved reached up to 2.65.

In brief, the optimized medium and other media with an identical initial pH of 7.0 were utilized for cultivating bacterial cells at a temperature of 30 °C for 48 h. The nutrient concentrations in the respective media are presented in Table 3. Bacterial inoculation was performed using similar OD_600_ values (1.0).

**Table 3:** Media used for bio-cementation experiments.

Name	Concentration of yeast extract (g/l)	Concentration of polypeptone (g/l)	Concentration of ammonium sulfate (g/l)	Predicted optical density (OD_600_)	Initial urease activity (mM/min)
Optimized medium	20	10	5	1.89	3.5
A medium	20	10	0	1.75	3.1
B medium	20	20	5	2.08	4.2
C medium	33.63	33.63	0	2.65	4.6

#### Biofilm growth

**Figure 5:**
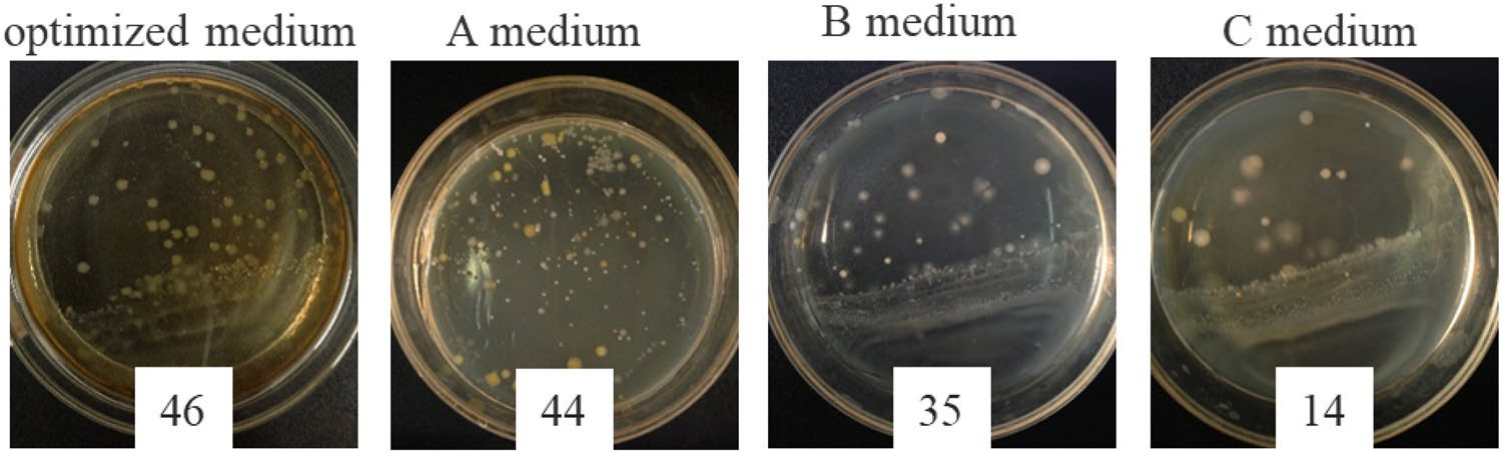
Images of the viable cell concentration method showing the biofilm growth (10^6^ times of dilution).

When an adequate supply of nutrients and oxygen is available, the concentration of suspended biomass increases, while the biofilm gradually develops. Consequently, higher availability of nutrient resources in the medium leads to an elevated growth rate of bacterial cell density and biofilm formation. Quantifying the content of biofilm poses challenges due to a lack of consensus among various techniques employed for its cultivation and study^41^. The plate colony counting method utilized in this study to determine CFU has certain limitations^36^, such as its inability to detect viable but non-culturable subpopulations within the biofilm. Nevertheless, this method remains widely used for estimating biofilm cell viability and can be implemented on most platforms. The viable cell concentration method was employed to demonstrate biofilm growth, as shown in Fig. 5.

The contents of biofilm in the samples treated with the optimized medium and A medium were similar (Fig. 5). The biofilm contents in the samples treated with the B medium decreased slightly. However, for the C medium, the contents of biofilm significantly decreased despite a much higher initial biomass concentration, which indicated that fewer bacterial cells participated in the biofilm formation. For A medium, the concentration of ammonium sulfate was smaller than that in the optimized medium, which might be the reason why the biofilm content of samples treated with A medium was a little smaller. The biofilm content of samples treated with the optimized medium was larger than that of the sample with B medium and the sample treated with C medium had the lowest biofilm content. It might be because the effect of polypeptone on biofilm growth was smaller than the effect of yeast extract on biofilm growth and increasing the concentration of polypeptone was not beneficial in promoting biofilm growth.

#### UCS and CaCO_3_ quantification

**Figure 6:**
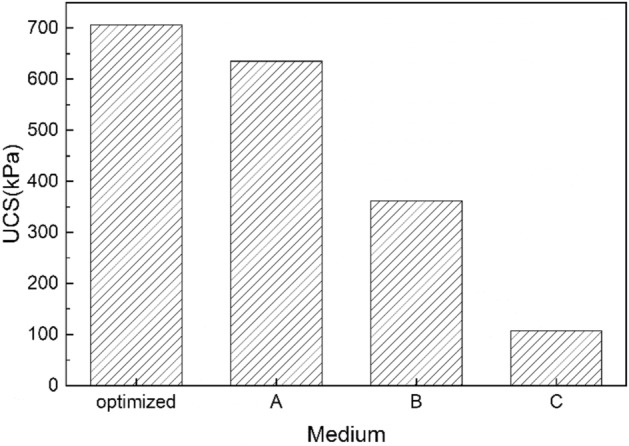
UCS of different solidified columns.

The UCS resulting from the utilization of different media is illustrated in Fig. 6. The sample treated with the optimized medium exhibited the highest strength (approximately 706 kPa), which was nearly twice as high as that observed for samples treated with B medium, and over 6 times greater than that of the sample treated with C medium (only 107 kPa). Although B medium and C medium contained more nutrients, their application resulted in poorer cementation effects, ultimately leading to lower strengths.

**Figure 7:**
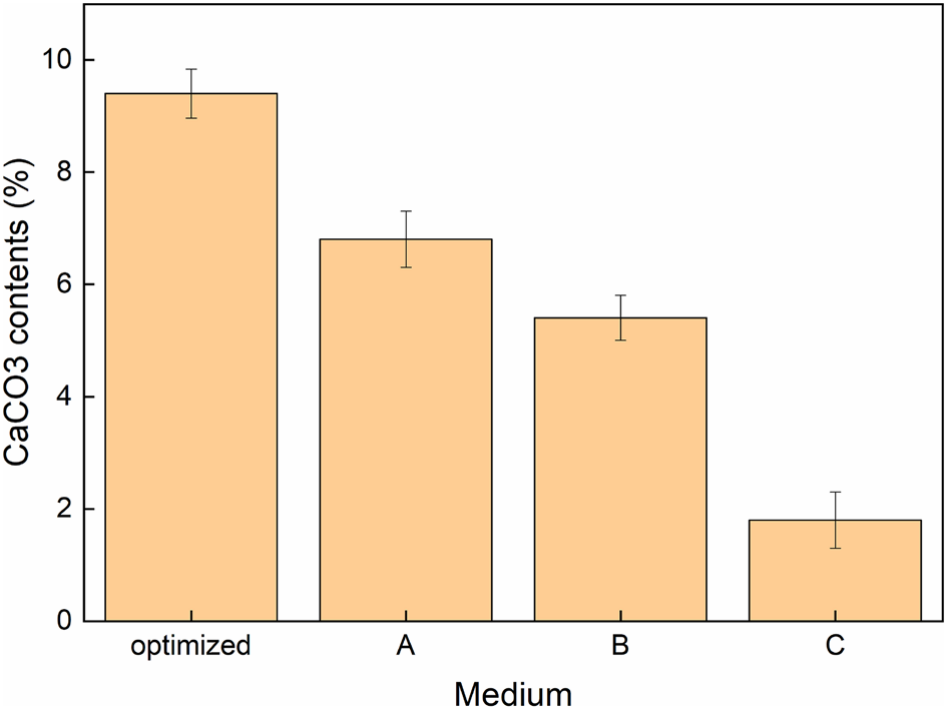
CaCO_3_ content in different solidified columns.

The CaCO_3_ content in the bio-cemented sand columns is illustrated in Fig. 7. The MICP technique yields varying levels of cementation at different heights of the bio-cemented soil columns^14,42^. With the exception of the C medium, samples treated with other media exhibited favorable cementation effects. This can be attributed to a lower participation of bacterial cells in biomass growth and CaCO_3_ production within the C medium, which aligns with the findings on biofilm contents. Notably, the sample treated with the optimized medium achieved almost 10% CaCO_3_ content, significantly higher than that observed in other columns. Previous studies have demonstrated that several factors influence both the type and amount of CaCO_3_ precipitation, including functional attributes of precipitating microorganisms, urea hydrolysis rate, dosages of urea and calcium ions, as well as amino acids like glutamic acid^30,43–46^. In our study, nutrient composition within the medium influenced biofilm growth and consequently affected CaCO_3_ precipitation.

### Limitations of the proposed RSM method

In this study, a robust prediction model was developed using RSM to enhance biofilm growth. However, according to a previous study^22^, the soil samples with different porosities need different biofilm growth rates; therefore, the RSM method should be correspondingly revised, which needs to be further studied. Moreover, several commonly used nutrients were considered in this RSM model, and various culture media were used in previous studies with different nutrients. The RSM model should be optimized in the future to meet the requirements of these different nutrients and various media. In addition, in this study, the dimension of sand columns was limited in the investigation of efficiency, despite the study's effectiveness in effectively demonstrating the benefits of the proposed RSM method for high efficiency and increased strengths in sand columns. It is still unknown how using the proposed RSM method for extensive soil improvement will turn out and the treatment should be carried out at a meter scale in the future study.

## Conclusions

The main challenge in the large-scale application of MICP lies in its low efficiency. In this study, a robust prediction model was developed using RSM to enhance biofilm growth. The prediction model demonstrated high accuracy and effectiveness, as evidenced by the close agreement between predicted and measured results. Furthermore, the optimized culture media (20.0 g/l yeast extract, 10.0 g/l polypeptone, 5.0 g/l ammonium sulfate, and 10.0 g/l NaCl) were obtained based on the prediction model.

Furthermore, sand solidification tests were conducted to evaluate the treatment effects of the optimized medium and verify the accuracy of the prediction model. The optimized medium, along with other media, was utilized for MICP treatment, and parameters such as UCS, biofilm growth, and CaCO_3_ contents were employed to assess the effectiveness of bio-cementation. Comparative results demonstrate that compared to other media, the optimized medium exhibits enhanced promotion of biofilm growth. Moreover, this study showcases the practicality of our prediction model as a platform technology that integrates culture medium to confer novel sensing capabilities and adjustive responsiveness onto structural materials. Additionally, our prediction model establishes a robust foundation for material manufacturing applications in civil and material engineering fields.

## Data Availability

Some or all data, models, or codes that support the findings of this study are available from the corresponding author upon reasonable request.
