# Author Correction: Optimization of culture medium to improve bio-cementation effect based on response surface method

**DOI:** 10.1038/s41598-024-61079-2

**Published:** 2024-05-03

**Authors:** Zhikun Pan, Shiding Cao

**Affiliations:** 1 Shenzhen SEZ Construction Solid Waste Resources Co. Ltd., Shenzhen, 518034 China; 2 State Key Laboratory for Tunnel Engineering, China University of Mining and Technology Beijing, Beijing, 100083 China; 3 Shenzhen General Integrated Transportation and Municipal Engineering Design and Research Institute Co. Ltd., Shenzhen, 518003 China

Correction to: *Scientific Reports* 10.1038/s41598-024-58063-1, published online 16 April 2024

In the original version of this Article, Zhikun Pan was incorrectly affiliated with ‘State Key Laboratory for Geomechanics and Deep Underground Engineering, China University of Mining and Technology, Beijing, 100083, China’ and ‘School of Mechanics and Civil Engineering, China University of Mining and Technology, Beijing, 100083, China’. The correct affiliation is listed below:

Shenzhen SEZ Construction Solid Waste Resources Co. Ltd., Shenzhen, 518034, China.

In addition, the original version of this Article omitted an affiliation for Shiding Cao. The correct affiliations are listed below.

State Key Laboratory for Tunnel Engineering, China University of Mining and Technology Beijing, Beijing, 100083, China

Shenzhen General Integrated Transportation and Municipal Engineering Design and Research Institute Co. Ltd., Shenzhen, 518003, China

The original Article has been corrected.

